# Response of high-risk MDS to azacitidine and lenalidomide is impacted by baseline and acquired mutations in a cluster of three inositide-specific genes

**DOI:** 10.1038/s41375-019-0416-x

**Published:** 2019-02-20

**Authors:** Matilde Y. Follo, Andrea Pellagatti, Richard N. Armstrong, Stefano Ratti, Sara Mongiorgi, Sara De Fanti, Maria Teresa Bochicchio, Domenico Russo, Marco Gobbi, Maurizio Miglino, Sarah Parisi, Giovanni Martinelli, Michele Cavo, Donata Luiselli, James A. McCubrey, Pann-Ghill Suh, Lucia Manzoli, Jacqueline Boultwood, Carlo Finelli, Lucio Cocco

**Affiliations:** 1 Cellular Signalling Laboratory, Human Anatomy Section, Department of Biomedical and Neuromotor Sciences, University of Bologna, Bologna, Italy; 2 Bloodwise Molecular Haematology Unit, Nuffield Division of Clinical Laboratory Sciences, Radcliffe Department of Medicine, University of Oxford, and Oxford BRC Haematology Theme, Oxford, UK; 3 Department of Biological, Geological, and Environmental Sciences, University of Bologna, Bologna, Italy; 4 Department of Hematology “L e A Seràgnoli”, University of Bologna, Bologna, Italy; 5 Chair of Hematology, Unit of Blood Disease and Stem Cell Transplantation, Department of Clinical and Experimental Sciences, University of Brescia, Brescia, Italy; 6 Department of Hematology and Oncology, University of Genova, Genova, Italy; 7 Istituto Scientifico Romagnolo per lo Studio e la Cura dei Tumori (IRST) IRCCS, Meldola, Italy; 8 Department of Cultural Heritage, University of Bologna, Ravenna, Italy; 9 Department of Microbiology and Immunology, Brody School of Medicine, East Carolina University, Greenville, NC USA; 10 School of Life Sciences, UNIST, Ulsan, Republic of Korea

**Keywords:** Cell signalling, Myelodysplastic syndrome, Targeted therapies

## Abstract

Specific myeloid-related and inositide-specific gene mutations can be linked to myelodysplastic syndromes (MDS) pathogenesis and therapy. Here, 44 higher-risk MDS patients were treated with azacitidine and lenalidomide and mutations analyses were performed at baseline and during the therapy. Results were then correlated to clinical outcome, overall survival (OS), leukemia-free-survival (LFS) and response to therapy. Collectively, 34/44 patients were considered evaluable for response, with an overall response rate of 76.25% (26/34 cases): 17 patients showed a durable response, 9 patients early lost response and 8 patients never responded. The most frequently mutated genes were ASXL1, TET2, RUNX1, and SRSF2. All patients early losing response, as well as cases never responding, acquired the same 3 point mutations during therapy, affecting respectively PIK3CD (D133E), AKT3 (D280G), and PLCG2 (Q548R) genes, that regulate cell proliferation and differentiation. Moreover, Kaplan–Meier analyses revealed that this mutated cluster was significantly associated with a shorter OS, LFS, and duration of response. All in all, a common mutated cluster affecting 3 inositide-specific genes is significantly associated with loss of response to azacitidine and lenalidomide therapy in higher risk MDS. Further studies are warranted to confirm these data and to further analyze the functional role of this 3-gene cluster.

## Introduction

Myelodysplastic syndromes (MDS) are a heterogeneous group of hematological malignancies characterized by peripheral blood cytopenia and a variable risk of evolution into acute myeloid leukemia (AML) [1]. MDS frequently show mutations in genes involved in RNA splicing (e.g., SF3B1, SRSF2, U2AF1, ZRSR2; approximately 40–60% of patients) [2, 3], DNA methylation (i.e., DNMT3A, TET2, IDH1, IDH2; approximately 30–40%) or chromatin remodelling (i.e., ASXL1, EZH2; approximately 15–25%), that are retrieved at diagnosis or can be specifically acquired during follow-up [4, 5]. For instance, not only ASXL1 mutations are associated with impaired hematopoiesis and are predictive of a poor outcome [6, 7], but also TP53, EZH2, SF3B1, and SRSF2 mutations have been recognized as being unfavorable for survival [8–10]. Moreover, specific gene mutations, such as those affecting TET2, have been associated with a favorable response to azacitidine [11], while the acquisition of TP53 or NRAS mutations has recently been related to resistance to lenalidomide [12, 13].

Epigenetic therapy is a first-line approach for MDS patients at higher risk of AML progression, as it delays the AML progression and improves the overall survival (OS) [14, 15]. Also lenalidomide is widely used in MDS patients, above all in those showing the deletion of the long arm of chromosome 5 [del(5q)]. However, for the MDS patients that are refractory or not suitable to the conventional strategies, the combination of these two drugs could be effective and is thus now clinically investigated [16, 17].

At a molecular level, azacitidine induces hypomethylation of critical genes implicated in cell proliferation and myeloid differentiation, such as those of the nuclear inositide signalling pathways [18]. On the other hand, lenalidomide inhibits cell proliferation by inducing the cereblon-dependent ubiquitination and degradation of casein kinase 1α [19]. Moreover, lenalidomide inhibits the activation of ERK and Akt-dependent pathways, inducing cell apoptosis and affecting the phosphatidylinositol (PI)-specific metabolism [20, 21].

Inositide-dependent signaling regulation is important in hematological malignancies [22–24]. Phosphoinositide-phospholipase C (PI-PLC) enzymes, such as PI-PLCgamma2 and PI-PLCbeta1, are important players of the signal transduction pathways [25–27]. Indeed, PI-PLCgamma2 is mapped on chromosome 16q23.3 and participates in cell proliferation and myeloid differentiation [28]. Also PI-PLCbeta1, which is localized on the 20p arm, is involved in cell cycle [29] and hematopoietic regulation, particularly in MDS [30, 31]. In fact, PI-PLCbeta1 increased expression has been associated with a positive response to azacitidine in MDS [32–35] and has also been inversely correlated to Akt activation [36]. Also Akt and PI3K-dependent signalling pathways play essential roles in hematological malignancies [37]. There are currently three known members of the Akt protein family, namely Akt1, Akt2, and Akt3, each encoded by a different gene [38]. These isoforms share a similar N-terminal Pleckstrin-homology (PH) domain and a central serine-threonine kinase domain, and their amino acid sequences are highly conserved [39]. Of note, isoform-specific Akt deregulation is frequently observed in different types of cancer. For instance, Akt3 impairment has been associated with multiple myeloma [40], and de novo Philadelphia chromosome-positive AML frequently show mutations of AKT3 and PIK3CD genes [41]. Indeed, the immune and leukocyte-restricted p110delta subunit of phosphatidylinositol-3-kinase (PI3K) [42], whose gene is PIK3CD, plays an important role in cell proliferation and has been proposed as a potential target in the treatment of AML [43, 44]. Moreover, recent studies showed that PIK3CD germline mutations in B-cells can lead to either gain or loss of function of PI3Kdelta, resulting in immune dysregulation [45–47].

Stemming from these data, in the present study we further investigated the role of azacitidine and lenalidomide therapy in MDS, focusing on the effect of the treatment on cancer myeloid and inositide-specific gene mutations. The acquisition of specific gene mutations during therapy might indeed be associated with MDS clinical outcome or therapy response, but it could also lead to a better comprehension of the mechanisms underlying the MDS pathogenesis and the effect of therapy.

## Methods

### Patient characteristics

Bone marrow (BM) and peripheral blood (PB) samples were obtained from 44 higher risk MDS patients [48, 49] who had given informed consent according to the Declaration of Helsinki (Table 1 and Supplementary Table 1). All samples came from several Italian hematological centers and were centralized at the Institute of Hematology “L. and A. Seràgnoli”, Policlinico Sant’Orsola–Malpighi Hospital, Bologna, Italy. Further details can be found in the Supplementary Information.

**Table 1:** Clinical, hematologic, and cytogenetic characteristics of the MDS patients

	Age	Sex	Diagnosis		Screening	Karyotype [no. metaphases with aberration]	Clinical outcome	Total cycles	Duration of therapy (months)	Time to first response (cycles)	Duration of response (months)	Survival (months)	Time to AML Evolution (months)	Cause of death
			WHO	WPSS										
^a^1	67	M	RAEB-2	VERY- HIGH	25/03/2013	COMPLEX	SD	10	10	NA	NA	14	8	AML
^a^2	67	F	RAEB-2	HIGH	02/04/2013	46, XX	CR	30	28	6	24	35	28	OVARIAN CANCER
^a^3	71	M	RAEB-2	HIGH	29/04/2013	47, XY, +8 [8]	mCR	38	36	4	10	41	36	AML, INFECTION
^a^4	76	F	RAEB-2	HIGH	13/05/2013	46, XX [1]	HI	8	8	4	3	12	9	AML, CACHEXIA
^a^5	68	M	RAEB-1	HIGH	13/05/2013	COMPLEX	SD	9	9	NA	NA	14		PNEUMONIA, CARDIAC FAILURE
^a^6	67	M	RAEB-2	HIGH	23/05/2013	46, XY	PR	10	10	2	5	30	10	AML
^a^7	72	M	RAEB-1	HIGH	26/06/2013	46, XY, del(7)(q22;34) [20]; del(7q31) [18]	SD	8	8	NA	NA	22	20	CEREBRAL HEMORRHAGE
8	82	M	RAEB-2	HIGH	28/06/2013	46, XY	CR	41	42	2	40	42		
9	67	F	RAEB-1	HIGH	01/07/2013	47, XX, +8	HI + mCR	12	18	2 (mCR); 5 (HI)	8 (HI); 16 (mCR)	42		
10	73	F	RAEB-1	INT	10/07/2013	45, X, del(X), del(20q)	HI + mCR	38	42	3 (HI) + 4 (mCR)	35 (HI); 34 (mCR)	42		
^a^11	75	F	RAEB-1	HIGH	10/07/2013	47, XX, +8	HI	20	21	1	19	28	21	DISEASE PROGRESSION
12	76	M	RAEB-1	INT	22/07/2013	46, XY	HI + mCR	8	8	1	5 (HI); 6 (mCR)	38		
^a^13	74	M	RAEB-2	ND	24/07/2013	ND	NA	1	1	NA	NA	2		PNEUMONIA
^a^14	78	M	RAEB-2	HIGH	05/08/2013	46, XY	HI	19	25	8	9	27	25	AML
^a^15	72	M	RAEB-2	HIGH	28/08/2013	46, XY, del(5), del(9)	NA	2	3	NA	NA	12		WORSENING OF CLINICAL CONDITIONS
16	70	M	RAEB-2	HIGH	29/08/2013	46, XY	CR	27	40	2	38	40		
^a^17	75	F	RAEB-2	HIGH	03/09/2013	46, XX, t(2;15)(q23;q26)	DP	2	2	NA	NA	5	2	AML
^a^18	72	F	RCMD- RS	HIGH	09/09/2013	COMPLEX	HI + mCR	13	14	2	11	16	15	AML, SEPSIS
^a^19	62	M	RAEB-1	HIGH	17/09/2013	46, XY, del(7), +X, [18]	CR	6	7	1	5	14		CARDIAC EVENT
^a^20	70	F	RAEB-2	HIGH	23/09/2013	46, XX	NA	2	2	NA	NA	2		COPD
^a^21	82	M	RCMD	HIGH	23/09/2013	47, XY, +8, −9,+3mar	HI	20	28	6	18	28		RESPIRATORY FAILURE
22	82	M	RAEB-2	HIGH	25/09/2013	46, XY, del(20q)	CR	36	39	1	38	39		
^a^23	68	M	RCMD	HIGH	03/10/2013	45, XY, del(7)	HI	3	3	1	2	3		SUDDEN DEATH
24	75	M	RAEB-2	VERY- HIGH	28/10/2013	45, XY, del(7), del(20)(q11)[3] / 46, XY [17]	NA	1	1	NA	NA	35		
^a^25	66	F	RAEB-2	HIGH	15/10/2013	46, XX	SD	8	8	NA	NA	14	14	AML
^a^26	77	M	RAEB-2	HIGH	30/10/2013	46, XXYY [5]	NA	1	1	NA	NA	5		HEART FAILURE
^a^27	48	F	RAEB-2	VERY- HIGH	06/11/2013	47, XY, + 8	CR	16	15	2	11	26		DISEASE PROGRESSION
^a^28	64	F	RAEB-1	INT	06/11/2013	46, XX	HI	10	10	5	3	12		PULMONARY CARCINOMA
^a^29	79	F	RAEB-2	ND	13/11/2013	ND	NA	2	2	NA	NA	3		HEART ATTACK
^a^30	66	M	RAEB-2	VERY- HIGH	15/11/2013	47, XY, +8	HI	9	9	3	4	10		WORSENING OF CLINICAL CONDITIONS
^a^31	75	F	RAEB-2	HIGH	22/11/2013	46, XX	HI	10	10	2	5	11	11	AML
^a^32	83	M	RAEB-2	HIGH	04/02/2014	47, XX, +8 [5]	SD	6	6	NA	NA	11	9	AML
^a^33	71	F	RAEB-2	VERY- HIGH	17/02/2014	COMPLEX	CR	7	7	2	5	12	9	AML, SEPSIS
^a^34	66	F	RAEB-2	VERY- HIGH	10/03/2014	COMPLEX	DP	1	1	NA	NA	2	1	AML, INFECTION
35	72	M	RAEB-1	VERY- HIGH	14/04/2014	COMPLEX	HI + mCR	11	11	4 (HI + mCR)	6 (HI)	32		
^a^36	69	M	RAEB-2	HIGH	19/05/2014	46, XY	mCR	6	6	2	3	16	13	AML
37	70	M	RAEB-1	HIGH	19/05/2014	46, XY, del(7q31)	SD	7	9	NA	NA	31		
^a^38	77	F	RAEB-2	VERY- HIGH	19/05/2014	COMPLEX	NA	2	4	NA	NA	4		BILATERAL PNEUMONIA
^a^39	82	F	RAEB-1	VERY- HIGH	04/08/2014	COMPLEX	NA	1	2	NA	NA	7	3	AML
^a^40	80	M	RAEB-2	VERY- HIGH	15/09/2014	46, XY, t(9,17) (p36;q23) [19]	NA	2	2	NA	NA	2		CACHEXIA, HEPATIC FAILURE
^a^41	78	M	RAEB-2	HIGH	19/08/2014	46, XY	HI + mCR	17	16	2 (HI); 2 (mCR)	14 (HI); 14 (mCR)	17		UNKNOWN
42	74	M	RAEB-2	VERY- HIGH	18/08/2014	46, XY, del(7) [1]	CR	26	28	1	27	28		
^a^43	66	M	RAEB-2	HIGH	20/10/2014	46, XY	NA	2	2	NA	NA	7		RESPIRATORY FAILURE
44	76	M	RAEB-2	HIGH	09/12/2014	46, XY	mCR	22	25	4	21	25		

*WHO* World Health Organization; *WPSS* WHO Prognostic Scoring System; *RAEB* refractory anemia with excess of blasts; *RCMD* refractory cytopenia with multilineage dysplasia; *RCMD-RS* refractory cytopenia with multilineage dysplasia and ringed sideroblasts; *VERY-HIGH* very-high-risk; *HIGH* high-risk; *INT* intermediate risk; *CR* complete remission; *mCR* marrow complete remission; *PR* partial remission; *HI* hematologic improvement; *SD* stable disease; *DP* disease progression; *AML* acute myeloid leukemia; *COPD* chronic obstructive pulmonary disease

^a^Patients deceased during follow-up

### Patient treatment and evaluation of response

Patients were treated with azacitidine (75 mg/m2/die for 7 days every 28 days) and lenalidomide (10 mg/day, days 1–21 or 6–21, orally) every 4 weeks. The response to treatment and the clinical outcome were evaluated according to the revised International Working Group (IWG) response criteria [50] (Table 1 and Supplementary Table 1). Further details can be found in the Supplementary Information.

### Isolation of mononuclear cells and genomic DNA extraction

For in vitro experiments, BM and PB mononuclear cells were isolated at the time of diagnosis and during the therapy, as described in the Supplementary Information.

### Illumina and Ion Torrent next-generation sequencing

The mutational profile of 32 recurrently mutated genes in myeloid malignancies was determined using an Illumina TruSeq Custom Amplicon next-generation sequencing gene panel (Supplementary Table 2) and the TruSeq Amplicon 2.0 BaseSpace app workflow [51] (Illumina, San Diego, CA, USA). 31 inositide-specific point mutations and small indels (Supplementary Table 3) were examined using the Ion Torrent S5 with an Ion AmpliSeq™ On-demand Panel (Thermo Fisher Scientific, Whaltam, MA, USA). Sequencing alignment was viewed by the Integrative Genomics Viewer Software (Broad Institute, Cambridge, MA, USA) using the Human Genome Build 19 (Hg19) as reference [52]. Further details can be found in the Supplementary Information.

### Statistical analyses

All statistical analyses were performed using the GraphPad Prism 5.0 Software (GraphPad Software, La Jolla, CA, USA), as described in the Supplementary Information.

## Results

### Patient outcomes

Between March 2013 and December 2017, forty-four patients diagnosed with high-risk MDS were treated with a combination of azacitidine and lenalidomide (Table 1). The median follow-up was 15 months (range 2–54 months). Thirty-one patients reached at least six cycles of therapy (T6) and were clinically evaluable for response. Moreover, three patients showed a disease progression or hematologic improvement before T4 and were evaluated for response too, so that 34 cases were clinically evaluated for response. According to the revised IWG criteria [50], the overall response rate (ORR) was 76.5% (26/34 cases): CR (8/34, 23.5%), PR (1/34, 2.9%), marrow CR (mCR, 3/34, 8.8%), HI (8/34, 23.5%), HI+mCR (6/34, 17.6%), whereas 6/34 patients (17.6%) had a stable disease and 2/34 cases (5.9%) had a disease progression. Among the patients evaluated for response, 13 patients showed a first positive response within T4 and maintained it at T8 and after (good responders, GR); 9 patients showed a positive response within T4 and lost response at T8 (transient responders, TR); 4 patients responded after T4 and maintained the response at T8 (late responders, LR); 8 patients never responded (non responders, NR).

### Illumina gene mutation analyses

Paired samples (pre- and post-treatment) were tested for mutations in genes that are recurrently mutated in myeloid malignancies. As the quality and quantity of DNA for each sample was critical, only 30/34 samples were tested at baseline and during the therapy (Table 2): at T4 (*n* = 2), T6 (*n* = 2), T7 (*n* = 2), T8 (*n* = 19) and T10 (*n* = 4), while 1 sample was tested at T4 and T8 (Table 2). Three of 30 patients showed no mutations either at baseline or during therapy (they were tested at baseline and T6, T7, and T10), while all other patients (27/30) had at least one mutation (Table 2). In this latter group of patients, two genes (NRAS and CEBPA), in two patients, acquired specific mutations only during the therapy, while all other genes were mutated in all 27 patients both at baseline and during the treatment, showing different VAFs between baseline and treatment. Remarkably, all samples showing a statistically significant decreasing VAF during therapy for all variants, as compared to baseline (*n* = 7), showed a favorable response to therapy (3 CR, 1 mCR, 1 HI+mCR, 1 PR, 1 HI). Conversely, none of the patients with SD (*n* = 7) showed a major decreasing VAF during therapy for all variants, as compared to baseline. All other patients had similar VAFs between baseline and therapy or showed a mixed behavior for all the variants identified (*n* = 13). Collectively, the most frequently mutated genes were ASXL1 (14 cases = 47%), TET2 (11 cases = 37%), RUNX1 (8 cases = 27%) and SRSF2 (5 cases = 17%). Interestingly, all patients showing the single SRSF2 mutation evolved to AML, while all patients without any somatic myeloid gene mutation had a favorable response (CR or HI) and did not progress into AML.

**Table 2:** Gene mutation analysis by Illumina cancer myeloid panel

Patient ID	Gene	Mutation type	VAF at T0	VAF at T4	VAF at T6	VAF at T7	VAF at T8	VAF at T10	T0 vs Therapy ***p < 0.01; **p < 0.05	Clinical outcome	AML evolution	Time to AML evolution (months)	Duration of response (months)
1	TP53	c.376-1G>A - splice acceptor	18,10	38,75					***	SD	YES	8	0
2	DNMT3A	P904L	15,68				16,63		ns	CR	YES	28	24
3	IDH2	R140Q	37,40				8,50		***	mCR	YES	36	10
	SRSF2	P95L	51,90				10,60		***				
	ASXL1	G646fs	32,00				9,90		***				
4	ASXL1	G646	12,10					20,30	***	HI	YES	9	5
	RUNX1	G135D	11,70					21,70	***				
	IDH1	R132C	0,32					21,20	***				
	KIT	E562*	2,40					5,90	***				
	SRSF2	P95L	4,80					29,10	***				
5	SF3B1	R625C		14,48			18,60		**	SD	YES	14	0
6	ASXL1	G644fs	10,60				5,70		***	PR	YES	10	6
	RUNX1	D133fs	10,60				4,50		***				
7	DNMT3A	R882C	30,10				44,90		***	SD	YES	20	0
	RUNX1	c.509-3C>G splice donor	14,50				43,30		***				
	NRAS	G12A	0				9,50		***				
8	ASXL1	L890F	7,12				13,39		***	CR	NO	0	52
9	SF3B1	K700E	35,10				39,00		**	HI + mCR	NO	0	16
	TET2	K306fs	27,80				38,20		***				
	TET2	L1360fs	26,70				38,20		***				
10	TET2	L264fs	25,40				30,50		**	HI + mCR	NO	0	47
	TET2	I1873T	25,80				30,00		**				
	PHF6	Y303*	42,00				46,90		***				
11	TET2	K875fs	11,80					14,20	**	HI	YES	21	19
	TET2	Y1245fs	8,40					12,10	**				
	SRSF2	p95H	9,80					5,40	**				
	ASXL1	G646fs	10,10					14,10	**				
	RUNX1	S322*	5,70					8,20	**				
12	ASXL1	G643fs	8,40				10,20		**	HI + mCR	NO	0	6
13	DNMT3A	R882C	19,80				21,70		**	HI	YES	25	9
	CBL	c.1096-7A>G Spice site	14,30				19,90		***				
	IDH2	R140Q	19,10				22,10		***				
	CEBPA	inframe TAD2	32,50				32,60		ns				
14	ASXL1	G635fs	27,30				14,90		***	CR	NO	0	50
15	TP53	c.97-2A>C - splice acceptor	50,03				3,10		***	HI + mCR	YES	15	11
16	DNMT3A	R882S	16,20				5,00		***	CR	NO	0	5
	IDH2	R140Q	18,10				3,60		***				
17	no somatic mutations								N/A	HI	NO	0	18
18	no somatic mutations								N/A	CR	NO	0	50
19	TET2	G1288fs	10,45	0,32					***	HI	NO	0	2
	TET2	R1451fs	19,13	14,52					***				
20	TET2	R1366H	50,10					48,80	ns	SD	YES	14	0
	SRSF2	P95H	9,30					18,80	***				
	SRSF2	H99N	5,20					9,40	***				
	SRSF2	P96FS	5,00					7,60	**				
	CEBPA	H219fs	0					10,40	***				
	CEBPA	S193fs	0					10,10	***				
	ASXL1	G646fs	13,00					15,10	**				
21	NRAS	G12V	18,72			15,57			**	HI	NO	0	3
	TET2	Q969fs	32,13			36,61			***				
	TET2	E1401*	31,99			38,24			***				
	SRSF2,MFSD11	P96fs	10,46			6,97			***				
	SRSF2,MFSD11	P95H	20,69			20,00			ns				
	CEBPA	P196dup	38,86			20,69			***				
	RUNX1	R157fs	31,07			34,95			**				
22	EZH2	S669R	44,90				32,10		***	HI	NO	0	5
	ASXL1	Y591fs	25,30				18,20		***				
	RUNX1	c.497_508+3dupGAAGTGGAAGAGGTA Splice region	12,00				9,20		**				
	ZRSR2	E65*	42,70				33,60		***				
	ZRSR2	E74*	3,10				5,30		***				
23	TET2	P1278Q	55,10				49,60		***	HI	YES	11	7
	U2AF1	S34F	5,60				9,50		***				
24	TET2	H650fs	35,83		33,74				**	SD	YES	9	0
	TET2	T1884A	38,51		37,21				ns				
	CBL	G397V	38,99		36,36				**				
	ASXL1	Q592*	37,62		35,75				**				
25	TP53	V73Argfs	12,31				1,74		***	CR	YES	9	5
26	TET2	E846*	8,90				7,00		**	mCR	YES	13	3
	SRSF2	P95H	7,80				6,20		ns				
	ASXL1	R693*	9,50				7,30		**				
27	TP53	H179Q	1,90				5,79		***	SD	NO	0	0
	ASXL1	G646fs	29,73				22,45		***				
	U2AF1	Q157P	38,97				25,41		***				
28	TET2	C1135Y	12,00				29,70		***	HI + mCR	NO	0	14
	TET2	Y1244fs	12,70				29,60		***				
	ASXL1	A640fs	12,50				30,40		***				
	RUNX1	N182fs	1,90				23,40		***				
29	no somatic mutations								N/A	CR	NO	0	32
30	NRAS	Y64C	1,14				6,26		***	mCR	NO	0	33
	TET2	P413fs	21,02				30,47		***				
	TET2	Q1507*	51,44				42,60		***				
	ASXL1	S1168fs	42,25				41,93		ns				
	RUNX1	P95T	41,99				43,73		**				

*VAF* variant allele frequency; *T0* baseline; *T4* 4th cycle therapy; *T6* 6th cycle therapy; *T7* 7th cycle therapy; *T8* 8th cycle therapy; *T10* 10th cycle therapy; *ns* not significant; *N/A* not applicable

***p <  0.01 vs T0; **p <  0.05 vs T0

### Inositide-specific gene mutation analyses

Paired samples (pre- and post-treatment) were also tested for other 31 genes, chosen among the inositide-specific signalling pathways (Supplementary Table 3). Eight genes showed no baseline mutations and did not acquire any variant during the therapy (PRKCA, GSK3A, GSK3B, MZF1, MYB, NFKB1, CDKN2B, and SLC29A2), whereas SOD2 and HFE genes showed no mutations at baseline but acquired variants during the therapy.

**Fig. 1:**
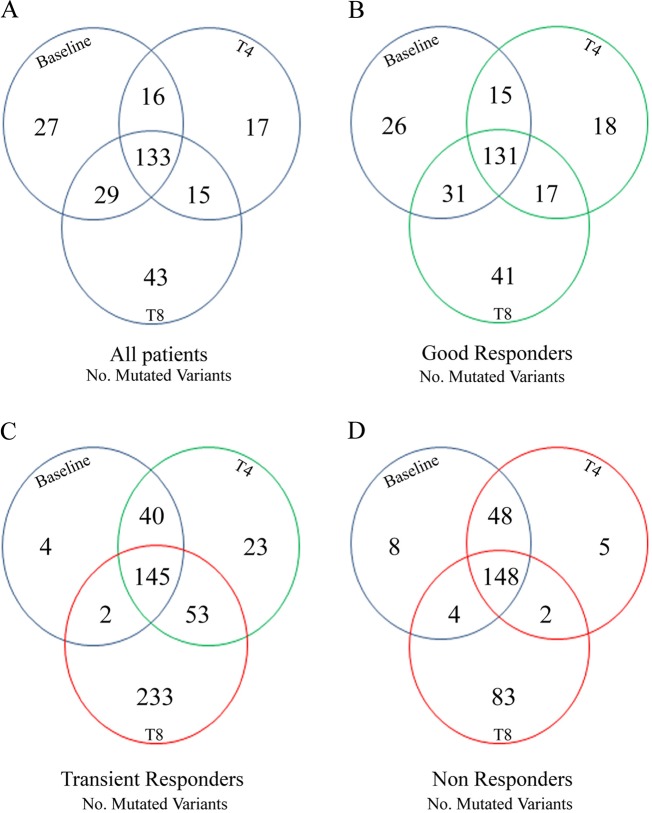
Inositide-Specific mutated variants in MDS patients at baseline, at the 4th cycle (T4) and at the 8th cycle (T8) of azacitidine and lenalidomide therapy. Venn diagram showing the global number of mutated variants in: **a** all patients analyzed, **b** Good Responders, **c** Transient Responders, **d** Non Responders

At baseline, 21/31 genes were mutated, with MAP2K3 gene showing 95 variants. During the treatment, 19/31 genes acquired specific variants: MTOR, PIK3CA, PIK3R1, TNF, SOD2, MAP2K1, PLCG2, MAP2K3, MAP2K2, PLCB1 at T4, and PIK3CD, MTOR, AKT3, MAP3K1, PIK3R1, HFE, CDKN1A, SOD2, AKT1, PLCG2, MAP2K3, MAP2K2, PIK3R2, PLCB1, PLCG1, RPS6KA3 at T8 (Supplementary Table 3). As shown in Fig. 1, in TR and NR patients, there was a significant increased VAF during therapy, as compared to baseline, with the acquisition of 233 specific variants at T8 in TR patients and 83 in NR patients. Interestingly, these two groups also showed a low number of variants at baseline: 4 in TR patients, affecting MAP2K2, CYPD26, and RPS6KA3 genes, and 8 in NR patients, affecting MAP3K1, PLCG2, MAP2K3, PLCB1 and RPS6KA3 genes. Therefore, VAF increased by 58 times in TR patients and 10 times in NR patients, as compared to baseline.

### Identification of a 3-gene cluster associated with loss of response

**Fig. 2:**
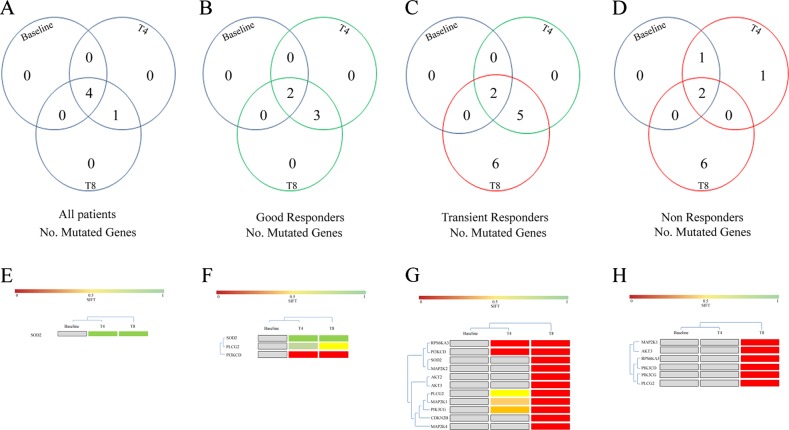
Inositide-Specific mutated genes according to the SIFT score in MDS patients at baseline, at the 4th cycle (T4) and at the 8th cycle (T8) of azacitidine and lenalidomide therapy. Venn diagram showing the global number of mutated genes, divided according to the SIFT score, in: **a** all patients analyzed, **b** Good Responders, **c** Transient Responders, **d** Non Responders. The bottom part of the Figure shows the genes acquiring specific mutations during the therapy in: **e** all patients analyzed (common T4 and T8, *n* = 1), **f** GR patients (common T4 and T8, *n* = 3), **g** TR patients (common T4 and T8, *n* = 5; T8 only, *n* = 6), **h** NR patients (T8 only, *n* = 6), clustered according to the SIFT score: gray squares indicate no mutation, green to red squares indicate a lower to higher probability of impaired protein function due to mutation

The SIFT score [53] was used to further analyze the mutation profile: 11 genes (6 only at T8 and 5 both at T4 and T8) were mutated in TR patients, while 7 genes (6 only at T8 and 1 only at T4) were mutated in NR patients (Fig. 2). On the contrary, in all patients responding to the treatment at T8 (including both GR and LR), only 3 genes (SOD2, PLCG2, PIK3CD) acquired specific common mutations at T4 and T8 (Fig. 2).

**Fig. 3:**
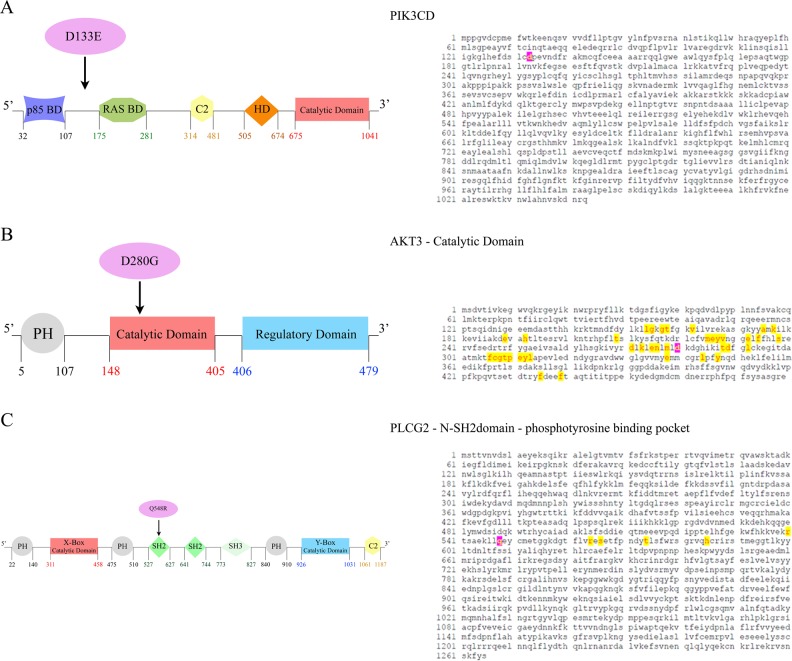
Common point mutations affecting 3 inositide-specific genes in MDS patients early losing response and never responding to azacitidine and lenalidomide therapy. Domain structure of **a** PIK3CD, **b** AKT3, and **c** PLCG2 proteins, along with the sequence domains affected by gene mutations: the mutated amino acids are highlighted in pink. Amino acids already known to be implicated in protein function are highlighted in yellow. Abbreviations: BD: binding domain; C2: calcium-binding domain; HD: hydrophobic regulatory domain; PH, Pleckstrin-homology domain; X-Box: phosphatidylinositol-specific phospholipase C X domain; SH2: Src homology 2 domain; SH3: Src homology 3 domain; Y-Box: phosphatidylinositol-specific phospholipase C Y domain

Interestingly, in both TR and NR patients, there was a common specific cluster of 6 mutated genes (MAP2K1, PIK3CD, RPS6KA3, AKT3, PIK3CG, PLCG2), detected as 88 variants in TR patients and 34 variants in NR patients (Fig. 2). A depth analysis of these variants not only showed that 3 genes (PIK3CD, AKT3, and PLCG2) were commonly altered at T8 in both these two groups but, more interestingly, the same 3 point mutations were acquired: D133E in PIK3CD gene, D280G in AKT3 gene, and Q548R in PLCG2 gene (Fig. 3). Interestingly, the sequence analysis of these point mutations revealed that the mutation affecting AKT3 was included in the catalytic domain of AKT3, while PLCG2 mutation was located within the N-terminal Src homology 2 (N-SH2) - phosphotyrosine binding pocket domain (Fig. 3).

### Survival analyses

**Fig. 4:**
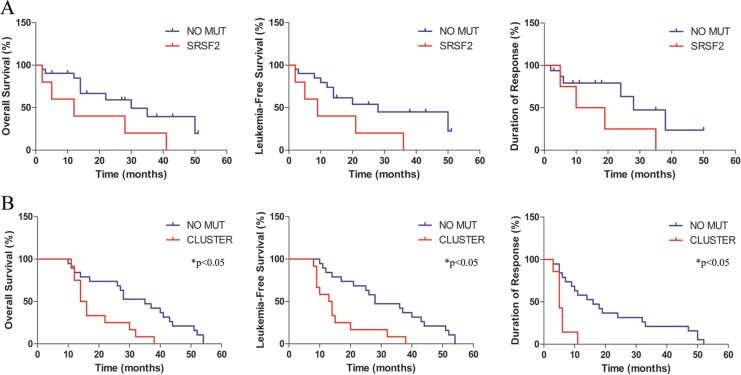
Kaplan–Meier estimates of overall survival, leukemia-free survival, and duration of response in MDS patients treated with azacitidine and lenalidomide. **a** Patients are stratified according to the presence of SRSF2 mutation (SRSF2) or the absence of SRSF2 mutation (NO MUT). **b** Patients are stratified according to the presence of our 3-inositide gene mutation (CLUSTER) or the absence of our 3-inositide gene mutation (NO MUT). *p < 0.05 CLUSTER vs NO MUT

As reported in Fig. 4, the association between SRSF2 mutations and OS was close to significant: 30 vs 12 months with 95% CI +2.15 to +2.84, *p* = 0.05; HR = 0.25 with 95% CI +0.06 to +1.04. Also the association between SRSF2 mutations and LFS was close to significant: 28 vs 9 months with 95% CI +2.76 to +3.45, *p* = 0.05; HR = 0.24 with 95% CI +0.06 to +1.02. On the contrary, SRSF2 mutations were not significantly associated with duration of response: 28 vs 14.5 months with 95% CI +1.64 to +2.21; *p* = 0.11; HR = 0.27 with 95% CI +0.06 to +1.35. On the other hand, the presence of our inositide-mutated 3-gene cluster was significantly associated with a shorter OS (35 vs 15 months with 95% CI +1.84 to +2.81, *p* = 0.046; HR = 0.24 with 95% CI +0.09 to +0.64), a shorter LFS (28 vs 13.5 months with 95% CI +1.58 to +2.56, *p* = 0.0011; HR = 0.19 with 95% CI +0.07 to +0.52) and a shorter duration of response (16 vs 5 months with 95% CI +2.78 to +3.62, *p* = 0.0012; HR = 0.09 with 95% CI +0.02 to +0.38).

## Discussion

Azacitidine alone is a standard therapy for MDS patients at higher risk of AML evolution, while lenalidomide is widely used in MDS with del(5q) deletion. The combination of azacitidine and lenalidomide has been clinically investigated, but the molecular effect of this treatment on mutation profiling is still not well known, although the extent of genetic rearrangements has been associated with outcome and response to treatment [54]. Moreover, as a reliable clinical evaluation of the combination therapy effect is possible only after several cycles of therapy, the identification of predictive molecular markers of response/resistance would be very helpful, above all because the persistence of mutated subclones can influence the response to epigenetic therapy [55].

Nuclear inositides are involved in hematopoietic differentiation and in MDS pathogenesis, genetically and epigenetically. Indeed, the presence of a PI-PLCbeta1 mono-allelic deletion in MDS patients has been associated with a higher probability of AML evolution [56]. On the other hand, PI-PLCbeta1 is also a specific target and a dynamic predictive marker of azacitidine effect, either positive or negative, in MDS patients [32, 33].

In this study we investigated the molecular mechanisms underlying azacitidine and lenalidomide therapy, focusing on the mutation profile of cancer myeloid genes (i.e., ASXL1, RUNX1, TET2, IDH1/2) as well as a selection of inositide-related genes, known to be involved in survival pathways (i.e., PI3K/Akt/mTOR, RAS/MAPK), hematopoietic differentiation (i.e., protein kinase C alpha, PI-PLCgamma2) [21], cell cycle (i.e., CDKN2B, protein kinase C alpha) [57] or drug metabolism (i.e., RPS6KA3, SOD2, CYP2D6, SLC29A2). In particular, we analyzed the mutation profile of MDS samples at baseline and during the combination therapy (possibly both at T4 and T8) and correlated it with clinical outcome, OS, LFS, and response to therapy.

In our patient cohort, three patients did not show any myeloid gene mutation neither at baseline nor during therapy: they all had a favorable response (CR or HI) and did not progress into AML. In contrast, all remaining patients showed at least one myeloid-related mutation at baseline and maintained it during the therapy, although showing different VAFs according to the clinical response. Indeed, all samples showing a decreasing VAF during therapy, as compared to baseline levels, had a favorable response (mainly CR, mCR or PR), while none of the nonresponders showed a decreasing VAF. Collectively, the most frequently mutated cancer myeloid genes were ASXL1 (47%), TET2 (37%), RUNX1 (27%) and SRSF2 (17%). Interestingly, all patients showing the single SRSF2 mutation evolved to AML, although the association between the presence of SRSF2 mutations and the LFS was close to significant (*p* = 0.05). This could be due to the low number of cases analyzed so far, but is still indicative of a negative correlation that needs to be investigated, also in the light of recent data from AML patients showing that SRSF2 persisting mutations are associated with a higher cumulative incidence of relapse [58].

As for the inositide-specific genes, 8 genes never showed mutations, neither at baseline nor during therapy: PRKCA, GSK3A, GSK3B, MZF1, MYB, NFKB1, CDKN2B, and SLC29A. Interestingly, this cluster included genes involved in inositide metabolism (PRKCA), cell cycle regulation (CDKN2B) and myeloid differentiation (MZF1 and MYB). On the other hand, 21/31 genes showed mutations at baseline and during the treatment, and 19/31 genes acquired specific variants. Collectively, there was a significant increased VAF during therapy in TR or NR patients, that showed an increased number of acquired mutations at T8. Moreover, these two groups also showed the lowest number of variants at baseline: 4 in TR patients, affecting MAP2K2, CYPD26, and RPS6KA3 genes, and 8 in NR patients, affecting MAP3K1, PLCG2, MAP2K3, PLCB1, and RPS6KA3 genes.

A deeper analysis of the inositide-specific mutation profile, performed by using the SIFT score [53], that predicts the effect of a point mutation on the protein function, revealed a small cluster of 6 genes commonly mutated only in TR and NR patients: MAP2K1, PIK3CD, RPS6KA3, AKT3, PIK3CG, and PLCG2. More interestingly, 3 of these genes (PIK3CD, AKT3, and PLCG2) acquired the same 3 point mutations: D133E in PIK3CD gene, D280G in AKT3 gene, and Q548R in PLCG2 gene. The analysis of the amino acid characteristics, as well as the examination of the protein sequence, revealed that D to E substitution is quite common, as both amino acids are quite frequently involved in protein active or binding sites and, in certain cases, they can also perform a similar role in the catalytic site of proteases or lipases. Moreover, this amino acid change, affecting PIK3CD gene, is located at the N-terminal and does not affect any known domain.

On the other hand, the D280G and Q548R mutations could affect amino acid polarization (thus possibly protein structure and function), in that D is negatively charged, G and Q have no charge and R is positively charged. Moreover, both AKT3 and PLCG2 point mutations localize within important protein domains: the catalytic domain of AKT3 and the N-terminal Src homology 2 (N-SH2)—phosphotyrosine binding pocket domain of PLCG2, although affecting amino acids not yet known to be important for protein function. Nevertheless, as both D and Q amino acids are quite frequently involved in protein active or binding sites, the mutations we retrieved might be associated with an impaired enzyme activity or protein stability. Indeed, the D to G substitution in AKT3 gene is usually a disfavoured one, in that it can affect protein conformation, increasing the conformational flexibility and using the G backbone to bind to phosphates. Moreover, AKT3 mutations affecting the catalytic kinase domain have been associated with elevated kinase activity in brain diseases [59], thus impacting the enzyme activity. Also the Q to R substitution is linked to protein stability, as both Q and R generally prefer to be on the surface of the proteins, but R make multiple hydrogen bonds with the phosphate, especially in SH2 domains [60].

Remarkably, the presence of our inositide-mutated 3-gene cluster (PIK3CD, AKT3, and PLCG2) was significantly associated with a shorter OS, a shorter LFS and a shorter duration of response, possibly predicting the unfavorable effect of azacitidine and lenalidomide combination therapy in MDS patients. Furthermore, as PIK3CD and AKT3 genes are actively involved in cell proliferation, it is likely that the acquisition of these specific point mutations in MDS not responding to therapy could give a proliferative advantage to mutated cells. On the other hand, as PLCG2 has been associated with myeloid differentiation, it is also likely that the acquisition of our specific point mutation in non responder MDS patients could result in an impaired hematopoietic differentiation that leads to a stable disease or AML progression.

All in all, our data confirm the results of previous studies [21, 22], in that also in this study inositides were associated with MDS. Here, PIK3CD, AKT3, and PLCG2 point mutations were correlated to and anticipated a negative clinical outcome, as all of the MDS patients included in this study that acquired the mutated cluster were also refractory to azacitidine and lenalidomide therapy at T8. Although this is a preliminary analysis, performed on a relatively small number of cases, the statistically significant association between this cluster and a shorter OS, LFS, and duration of response pave the way to larger studies.

To our knowledge, this is the first time that a systematic mutation analysis of inositide-related genes during azacitidine and lenalidomide therapy in MDS has been performed. More importantly, our findings indicate that a specific mutated 3-gene cluster is associated with early loss of response or refractoriness.

Given the involvement of nuclear inositides in cell cycle and in hematopoietic differentiation, and on the basis of our results, we feel that further investigating the effect of these point mutations on protein function could be important to understand the basic and translational implications of these mutations, to find alternative strategies aiming to activate specific signalling pathways to induce cancer cell apoptosis and/or normal myeloid differentiation in MDS.

## Supplementary information

Supplementary Information

Supplementary Table 1

Supplementary Table 2

Supplementary Table 3

## References

[1] Prebet T, Zeidan A (2016). Trends in clinical investigation for myelodysplastic syndromes. Clin Lymphoma Myeloma Leuk.

[2] Armstrong RN, Steeples V, Singh S, Sanchi A, Boultwood J, Pellagatti A (2018). Splicing factor mutations in the myelodysplastic syndromes: target genes and therapeutic approaches. Adv Biol Regul.

[3] Pellagatti A, Boultwood J (2017). Splicing factor gene mutations in the myelodysplastic syndromes: impact on disease phenotype and therapeutic applications. Adv Biol Regul.

[4] Stosch JM, Heumuller A, Niemoller C, Bleul S, Rothenberg-Thurley M, Riba J (2018). Gene mutations and clonal architecture in myelodysplastic syndromes and changes upon progression to acute myeloid leukaemia and under treatment. Br J Haematol.

[5] Kennedy JA, Ebert BL (2017). Clinical implications of genetic mutations in myelodysplastic syndrome. J Clin Oncol.

[6] Uni M, Masamoto Y, Sato T, Kamikubo Y, Arai S, Hara E (2018). Modeling ASXL1 mutation revealed impaired hematopoiesis caused by derepression of p16Ink4a through aberrant PRC1-mediated histone modification. Leukemia.

[7] Mangaonkar AA, Gangat N, Al-Kali A, Elliott MA, Begna KH, Hanson CA (2018). Prognostic impact of ASXL1 mutations in patients with myelodysplastic syndromes and multilineage dysplasia with or without ring sideroblasts. Leuk Res.

[8] Gangat N, Mudireddy M, Lasho TL, Finke CM, Nicolosi M, Szuber N (2018). Mutations and prognosis in myelodysplastic syndromes: karyotype-adjusted analysis of targeted sequencing in 300 consecutive cases and development of a genetic risk model. Am J Hematol.

[9] Pellagatti A, Armstrong RN, Steeples V, Sharma E, Repapi E, Singh S (2018). Impact of spliceosome mutations on RNA splicing in myelodysplasia: dysregulated genes/pathways and clinical associations. Blood.

[10] Arbab Jafari Pourya, Ayatollahi Hossein, Sadeghi Ramin, Sheikhi Maryam, Asghari Amir (2018). Prognostic significance of SRSF2 mutations in myelodysplastic syndromes and chronic myelomonocytic leukemia: a meta-analysis. Hematology.

[11] Unnikrishnan A, Papaemmanuil E, Beck D, Deshpande NP, Verma A, Kumari A (2017). Integrative genomics identifies the molecular basis of resistance to azacitidine therapy in myelodysplastic syndromes. Cell Rep.

[12] Jadersten M, Saft L, Smith A, Kulasekararaj A, Pomplun S, Gohring G (2011). TP53 mutations in low-risk myelodysplastic syndromes with del(5q) predict disease progression. J Clin Oncol.

[13] da Silva-Coelho P, Kroeze LI, Yoshida K, Koorenhof-Scheele TN, Knops R, van de Locht LT (2017). Clonal evolution in myelodysplastic syndromes. Nat Commun.

[14] Steensma DP (2018). Myelodysplastic syndromes current treatment algorithm 2018. Blood Cancer J.

[15] Finelli C, Follo MY, Stanzani M, Parisi S, Clissa C, Mongiorgi S (2016). Clinical impact of hypomethylating agents in the treatment of myelodysplastic syndromes. Curr Pharm Des.

[16] Sekeres MA, Othus M, List AF, Odenike O, Stone RM, Gore SD (2017). Randomized phase II study of azacitidine alone or in combination with lenalidomide or with vorinostat in higher-risk myelodysplastic syndromes and chronic myelomonocytic leukemia: North American Intergroup Study SWOG S1117. J Clin Oncol.

[17] Sekeres MA, Tiu RV, Komrokji R, Lancet J, Advani AS, Afable M (2012). Phase 2 study of the lenalidomide and azacitidine combination in patients with higher-risk myelodysplastic syndromes. Blood.

[18] Mongiorgi S, Follo MY, Yang YR, Ratti S, Manzoli L, McCubrey JA (2016). Selective activation of nuclear PI-PLCbeta1 during normal and therapy-related differentiation. Curr Pharm Des.

[19] Fink EC, Ebert BL (2015). The novel mechanism of lenalidomide activity. Blood.

[20] Kotla V, Goel S, Nischal S, Heuck C, Vivek K, Das B (2009). Mechanism of action of lenalidomide in hematological malignancies. J Hematol Oncol.

[21] Poli A, Ratti S, Finelli C, Mongiorgi S, Clissa C, Lonetti A (2018). Nuclear translocation of PKC-alpha is associated with cell cycle arrest and erythroid differentiation in myelodysplastic syndromes (MDSs). FASEB J.

[22] Mongiorgi S, Finelli C, Yang YR, Clissa C, McCubrey JA, Billi AM (2016). Inositide-dependent signaling pathways as new therapeutic targets in myelodysplastic syndromes. Expert Opin Ther Targets.

[23] Follo MY, Mongiorgi S, Finelli C, Clissa C, Ramazzotti G, Fiume R (2010). Nuclear inositide signaling in myelodysplastic syndromes. J Cell Biochem.

[24] Faenza I, Billi AM, Follo MY, Fiume R, Martelli AM, Cocco L (2005). Nuclear phospholipase C signaling through type 1 IGF receptor and its involvement in cell growth and differentiation. Anticancer Res.

[25] Ramazzotti G, Faenza I, Fiume R, Matteucci A, Piazzi M, Follo MY (2011). The physiology and pathology of inositide signaling in the nucleus. J Cell Physiol.

[26] Cocco L, Follo MY, Manzoli L, Suh PG (2015). Phosphoinositide-specific phospholipase C in health and disease. J Lipid Res.

[27] Ratti S, Mongiorgi S, Ramazzotti G, Follo MY, Mariani GA, Suh PG (2017). Nuclear inositide signaling Via phospholipase C. J Cell Biochem.

[28] Barbosa CM, Bincoletto C, Barros CC, Ferreira AT, Paredes-Gamero EJ (2014). PLCgamma2 and PKC are important to myeloid lineage commitment triggered by M-SCF and G-CSF. J Cell Biochem.

[29] Ratti S, Ramazzotti G, Faenza I, Fiume R, Mongiorgi S, Billi AM (2018). Nuclear inositide signaling and cell cycle. Adv Biol Regul.

[30] Manzoli L, Mongiorgi S, Clissa C, Finelli C, Billi AM, Poli A (2014). Strategic role of nuclear inositide signalling in myelodysplastic syndromes therapy. Mini Rev Med Chem.

[31] Mongiorgi S, Follo MY, Clissa C, Giardino R, Fini M, Manzoli L (2012). Nuclear PI-PLC beta1 and myelodysplastic syndromes: from bench to clinics. Curr Top Microbiol Immunol.

[32] Cocco L, Finelli C, Mongiorgi S, Clissa C, Russo D, Bosi C (2015). An increased expression of PI-PLCbeta1 is associated with myeloid differentiation and a longer response to azacitidine in myelodysplastic syndromes. J Leukoc Biol.

[33] Fili C, Malagola M, Follo MY, Finelli C, Iacobucci I, Martinelli G (2013). Prospective phase II Study on 5-days azacitidine for treatment of symptomatic and/or erythropoietin unresponsive patients with low/INT-1-risk myelodysplastic syndromes. Clin Cancer Res.

[34] Follo MY, Russo D, Finelli C, Mongiorgi S, Clissa C, Fili C (2012). Epigenetic regulation of nuclear PI-PLCbeta1 signaling pathway in low-risk MDS patients during azacitidine treatment. Leukemia.

[35] Follo MY, Finelli C, Mongiorgi S, Clissa C, Bosi C, Testoni N (2009). Reduction of phosphoinositide-phospholipase C beta1 methylation predicts the responsiveness to azacitidine in high-risk MDS. Proc Natl Acad Sci USA.

[36] Follo MY, Finelli C, Mongiorgi S, Clissa C, Chiarini F, Ramazzotti G (2011). Synergistic induction of PI-PLCbeta1 signaling by azacitidine and valproic acid in high-risk myelodysplastic syndromes. Leukemia.

[37] Ricciardi MR, Mirabilii S, Licchetta R, Piedimonte M, Tafuri A (2017). Targeting the Akt, GSK-3, Bcl-2 axis in acute myeloid leukemia. Adv Biol Regul.

[38] Tang Y, Jiang Z, Luo Y, Zhao X, Wang L, Norris C (2014). Differential effects of Akt isoforms on somatic cell reprogramming. J Cell Sci.

[39] Yang ZZ, Tschopp O, Baudry A, Dummler B, Hynx D, Hemmings BA (2004). Physiological functions of protein kinase B/Akt. Biochem Soc Trans.

[40] Li QY, Chen L, Hu N, Zhao H (2018). Long non-coding RNA FEZF1-AS1 promotes cell growth in multiple myeloma via miR-610/Akt3 axis. Biomed Pharmacother.

[41] Konoplev S, Yin CC, Kornblau SM, Kantarjian HM, Konopleva M, Andreeff M (2013). Molecular characterization of de novo Philadelphia chromosome-positive acute myeloid leukemia. Leuk Lymphoma.

[42] Okkenhaug K, Vanhaesebroeck B (2003). PI3K in lymphocyte development, differentiation and activation. Nat Rev Immunol.

[43] Xie C, He Y, Zhen M, Wang Y, Xu Y, Lou L (2017). Puquitinib, a novel orally available PI3Kdelta inhibitor, exhibits potent antitumor efficacy against acute myeloid leukemia. Cancer Sci.

[44] Sujobert P, Bardet V, Cornillet-Lefebvre P, Hayflick JS, Prie N, Verdier F (2005). Essential role for the p110delta isoform in phosphoinositide 3-kinase activation and cell proliferation in acute myeloid leukemia. Blood.

[45] Compagno M, Wang Q, Pighi C, Cheong TC, Meng FL, Poggio T (2017). Phosphatidylinositol 3-kinase delta blockade increases genomic instability in B cells. Nature.

[46] Avery DT, Kane A, Nguyen T, Lau A, Nguyen A, Lenthall H (2018). Germline-activating mutations in PIK3CD compromise B cell development and function. J Exp Med.

[47] Dornan GL, Siempelkamp BD, Jenkins ML, Vadas O, Lucas CL, Burke JE (2017). Conformational disruption of PI3Kdelta regulation by immunodeficiency mutations in PIK3CD and PIK3R1. Proc Natl Acad Sci USA.

[48] Vardiman JW, Harris NL, Brunning RD (2002). The World Health Organization (WHO) classification of the myeloid neoplasms. Blood.

[49] Greenberg PL, Tuechler H, Schanz J, Sanz G, Garcia-Manero G, Sole F (2012). Revised international prognostic scoring system for myelodysplastic syndromes. Blood.

[50] Cheson BD, Greenberg PL, Bennett JM, Lowenberg B, Wijermans PW, Nimer SD (2006). Clinical application and proposal for modification of the International Working Group (IWG) response criteria in myelodysplasia. Blood.

[51] Pellagatti A, Roy S, Di Genua C, Burns A, McGraw K, Valletta S (2016). Targeted resequencing analysis of 31 genes commonly mutated in myeloid disorders in serial samples from myelodysplastic syndrome patients showing disease progression. Leukemia.

[52] Thorvaldsdottir H, Robinson JT, Mesirov JP (2013). Integrative Genomics Viewer (IGV): high-performance genomics data visualization and exploration. Brief Bioinform.

[53] Ng PC, Henikoff S (2003). SIFT: Predicting amino acid changes that affect protein function. Nucleic Acids Res.

[54] Ganster C, Shirneshan K, Salinas-Riester G, Braulke F, Schanz J, Platzbecker U (2015). Influence of total genomic alteration and chromosomal fragmentation on response to a combination of azacitidine and lenalidomide in a cohort of patients with very high risk MDS. Leuk Res.

[55] Uy GL, Duncavage EJ, Chang GS, Jacoby MA, Miller CA, Shao J (2017). Dynamic changes in the clonal structure of MDS and AML in response to epigenetic therapy. Leukemia.

[56] Follo MY, Finelli C, Clissa C, Mongiorgi S, Bosi C, Martinelli G (2009). Phosphoinositide-phospholipase C beta1 mono-allelic deletion is associated with myelodysplastic syndromes evolution into acute myeloid leukemia. J Clin Oncol.

[57] Poli A, Mongiorgi S, Cocco L, Follo MY (2014). Protein kinase C involvement in cell cycle modulation. Biochem Soc Trans.

[58] Rothenberg-Thurley M, Amler S, Goerlich D, Kohnke T, Konstandin NP, Schneider S (2018). Persistence of pre-leukemic clones during first remission and risk of relapse in acute myeloid leukemia. Leukemia.

[59] Alcantara D, Timms AE, Gripp K, Baker L, Park K, Collins S (2017). Mutations of AKT3 are associated with a wide spectrum of developmental disorders including extreme megalencephaly. Brain.

[60] Betts MJ, Russell RB. Amino Acid Properties and Consequences of Substitutions. In: Barnes MR, Gray IC, editors. Bioinformatics for Geneticists: John Wiley & Sons; 2003. p. 289-316.

