# Supplementary material for: Response of high-risk MDS to azacitidine and lenalidomide is impacted by baseline and acquired mutations in a cluster of three inositide-specific genes
Source: Leukemia. 2019 Feb 20;33(9):2276–90. doi: 10.1038/s41375-019-0416-x (PMC6733710; doi:10.1038/s41375-019-0416-x)
Supplement: Supplementary file 1 — Supplementary Information [file 41375_2019_416_MOESM1_ESM.pdf]

SUPPLEMENTARY INFORMATION: MATERIALS AND METHODS

Patient Characteristics. All samples came from several Italian hematological centers and were centralized at the Institute of Hematology "L. and A. Seràgnoli", Policlinico Sant'Orsola–Malpighi Hospital, Bologna, Italy. The hematological centres involved were: the Haematology and Haematopoietic Stem Cell Transplant Center, S.Salvatore Hospital, Pesaro, Italy; the Hematology Unit, Arcispedale Santa Maria Nuova IRCCS, Reggio Emilia, Italy; the Division of Hematology, Guglielmo da Saliceto Hospital, Piacenza, Italy; the Hematology Unit, Hospital Santa Maria delle Croci, Ravenna, Italy; the Division of Hematology and Bone Marrow Transplantation, Udine, Italy; the Hematology and BMT Center, Department Medicine and Surgery, University of Parma, Parma, Italy; the Department of Medical and Surgical Sciences, University of Modena and Reggio Emilia, Modena, Italy; the Hematology Section, Department of Medical Sciences, University of Ferrara, Ferrara, Italy; and the Hematology Unit, Infermi Hospital, Rimini, Italy. The MDS diagnosis was defined according to the World Health Organization (WHO) classification [1] and, according to the Revised International prognostic scoring system (R-IPSS) [2], patients were divided into subgroups by risk: intermediate risk (n=8), high-risk (n=16), very high risk (n=18) and non determined (n=2, patients with RAEB-2). However, throughout the text, all patients with MDS are defined as high-risk MDS.

Patient treatment and evaluation of response. Patients were treated with Azacitidine (75 mg/m2/die for 7 days every 28 days) and Lenalidomide (10 mg/day, days 1-21 or 6-21, orally) every 4 weeks. The induction treatment was planned for 8 cycles. For responder patients this schedule was continued until disease progression or unacceptable toxicity. The response to treatment and the clinical outcome were evaluated according to the revised International Working Group (IWG) response criteria [3]. Patients were considered evaluable if they completed at least 6 cycles of therapy or showed either a positive response or disease progression before the 6th cycle

(T6). We also recorded the time to AML evolution (calculated from the date of diagnosis according to the WHO classification [1] , i.e. >20% marrow blasts), survival and causes of death (Table 1). Data were censored when patients died or were lost during follow-up. Patients who achieved a complete remission (CR), partial remission (PR), or any hematologic improvement (HI), according to the revised IWG criteria [3], were considered responders, whereas all the other outcomes were defined as non responders. The duration of response was assessed in patients who showed a clinical response to treatment.

Isolation of Mononuclear Cells and Genomic DNA Extraction. BM and PB mononuclear cells (MNCs) were isolated by Ficoll-Paque density-gradient centrifugation (Amersham Biosciences, Uppsala, Sweden), according to the manufacturer's instructions. All analyses were performed on samples from patients at the time of diagnosis and during the therapy. MNCs from healthy subjects were also extracted, and used as an internal control. Genomic DNA was isolated from total MNCs by using the QIAamp DNA Blood Mini Kit (Qiagen Ltd) according to the manufacturer's instructions.

Illumina Next Generation Sequencing. The mutational profile of the MDS patients was determined using a TruSeq Custom Amplicon next-generation sequencing gene panel (32 recurrently mutated genes in myeloid malignancies, Supplementary Table 2) and TruSeq Amplicon 2.0 BaseSpace app workflow [4]. Briefly, amplicon libraries were generated from 250ng of genomic DNA prior to 2x150bp paired-end sequencing on a Miseq platform. Variants were determined using Somatic Variant Caller (Illumina) prior to annotation and filtering using Illumina VariantStudio v.2.2 with criteria previously described [4].

Ion Torrent Next Generation Sequencing. Inositide-specific point mutations and small indels were examined using the Ion Torrent S5 with an Ion AmpliSeq™ On-demand Panel designed to analyze

31 inositide-specific genes (Thermo Fisher Scientific). Successful sequencing required a minimum of 10 ng DNA. Library construction was performed using the Ion AmpliSeq™ Library Kit (Thermo Fisher Scientific). The library was quantified using the Ion Libray TaqMan TM Quantitation Kit (Thermo Fisher Scientific) after ligation with the Ion Xpress TM Barcode Adapters (Thermo Fisher Scientific), following manufacturer's instructions. Sequence alignment and analysis were performed using the Ion Torrent Suite Software v.5.8.0 and the Ion Reporter software v.5.10.2.0 (Thermo Fisher Scientific). Sequencing alignment was viewed by the Integrative Genomics Viewer Software (Broad Institute, Cambridge, MA, USA) using Human Genome Build 19 (Hg19) as the reference [5]. A minimum coverage depth per amplicon of 250 was required; variant frequency of 10% and higher was considered positive. Somatic non-synonymous mutations were reported. Raw sequence data were available to reanalyze sequencing reads for possible amplifications, comparisons between paired samples, and confirmation of negative results in regions of interest.

Statistical Analyses. All statistical analyses were performed using the GraphPad Prism 5.0 Software (GraphPad Software, La Jolla, CA, USA). The difference between variant allele frequencies (VAFs) at baseline and during therapy was analyzed by a Two-Way Analysis of Variance Test with the Bonferroni post-test. All values with $p<0.05$ were considered statistically significant. Gene mutations were also analyzed by gene frequency, loci altered, pathways involved and correlated to clinical parameters, including OS, leukemia-free survival (LFS) and response to treatment. Survival analyses were done according to the Kaplan-Meier method and compared using the Log-rank test.

REFERENCES

1. Vardiman JW, Harris NL, Brunning RD. The World Health Organization (WHO) classification of the myeloid neoplasms. *Blood* 2002 Oct 1; **100**(7)**:** 2292-2302.

2. Greenberg PL, Tuechler H, Schanz J, Sanz G, Garcia-Manero G, Sole F, *et al.* Revised international prognostic scoring system for myelodysplastic syndromes. *Blood* 2012 Sep 20; **120**(12)**:** 2454-2465.

3. Cheson BD, Greenberg PL, Bennett JM, Lowenberg B, Wijermans PW, Nimer SD, *et al.* Clinical application and proposal for modification of the International Working Group (IWG) response criteria in myelodysplasia. *Blood* 2006 Jul 15; **108**(2)**:** 419-425.

4. Pellagatti A, Roy S, Di Genua C, Burns A, McGraw K, Valletta S, *et al.* Targeted resequencing analysis of 31 genes commonly mutated in myeloid disorders in serial samples from myelodysplastic syndrome patients showing disease progression. *Leukemia* 2016 Jan; **30**(1)**:** 247-250.

5. Thorvaldsdottir H, Robinson JT, Mesirov JP. Integrative Genomics Viewer (IGV): high-performance genomics data visualization and exploration. *Brief Bioinform* 2013 Mar; **14**(2)**:** 178-192.
