# Supplementary material for: Response of high-risk MDS to azacitidine and lenalidomide is impacted by baseline and acquired mutations in a cluster of three inositide-specific genes
Source: Leukemia. 2019 Feb 20;33(9):2276–90. doi: 10.1038/s41375-019-0416-x (PMC6733710; doi:10.1038/s41375-019-0416-x)
Supplement: Supplementary file 2 — Supplementary Table 1 [file 41375_2019_416_MOESM2_ESM.pdf]

Supplementary Table 1. Summarized patients' characteristics

Characteristics	
**General**	
No. patients	44
Male/female ratio	27:17
Median age, years (range)	72 (48-83)
**Cytogenetic Categories (WPSS)**	
Good	17
Intermediate	11
Poor	14
Not Determined	2
**Cytogenetic Categories in more detail**	
Normal	15
Del(20q)	2
Monosomy 7	6
Trisomy 8	7
Complex (More than 3 chromosomal aberrations)	8
Unknown	2
Other	4
