# Supplementary material for: Response of high-risk MDS to azacitidine and lenalidomide is impacted by baseline and acquired mutations in a cluster of three inositide-specific genes
Source: Leukemia. 2019 Feb 20;33(9):2276–90. doi: 10.1038/s41375-019-0416-x (PMC6733710; doi:10.1038/s41375-019-0416-x)
Supplement: Supplementary file 3 — Supplementary Table 2 [file 41375_2019_416_MOESM3_ESM.pdf]

Supplementary Table 2. List of genes analyzed by Illumina TruSeq Custom Amplicon Next-Generation Sequencing

ASXL1	*HRAS*	*PTEN*
ATRX	*IDH1*	*RUNX1*
CBL	*IDH2*	*SETBP1*
CBLB	*JAK2*	*SF3B1*
CBLC	*KIT*	*SRSF2*
CEBPA	*KRAS*	*TET2*
CSF3R	*MPL*	*TP53*
DNMT3A	*NPM1*	*U2AF1*
ETV6	*NRAS*	*WT1*
EZH2	*PDGFRA*	*ZRSR2*
FLT3	*PHF6*	
