# Supplementary material for: Response of high-risk MDS to azacitidine and lenalidomide is impacted by baseline and acquired mutations in a cluster of three inositide-specific genes
Source: Leukemia. 2019 Feb 20;33(9):2276–90. doi: 10.1038/s41375-019-0416-x (PMC6733710; doi:10.1038/s41375-019-0416-x)
Supplement: Supplementary file 4 — Supplementary Table 3 [file 41375_2019_416_MOESM4_ESM.pdf]

Supplementary Table 3. List of Genes analyzed by Ion Torrent and showing mutations at Baseline (T0), 4th Cycle (T4) or 8th Cycle (T8) of Azaciotidine and Lenalidomide Therapy

	**All Genes**	**Genes mutated at T0**	**Genes mutated at T4**	**Genes mutated at T8**
1	AKT1	PIK3CD	PIK3CD	PIK3CD
2	AKT2	MTOR	MTOR	MTOR
3	AKT3	MTOR|MTOR-AS1	MTOR|MTOR-AS1	MTOR|MTOR-AS1
4	MAP2K1	AKT3	AKT3	AKT3
5	MAP2K2	PIK3CA	PIK3CA	PIK3CA
6	MAP2K3	MAP3K1	MAP3K1	MAP3K1
7	MAP2K4	PIK3R1	PIK3R1	PIK3R1
8	MAP3K1	CDKN1A	CDKN1A	CDKN1A
9	PLCB1	AKT1	AKT1	AKT1
10	PLCG1	AKT2	AKT2	AKT2
11	PLCG2	PLCG2	PLCG2	PLCG2
12	PIK3CA	MAP2K4	MAP2K4	MAP2K4
13	PIK3CD	MAP2K3	MAP2K3	MAP2K3
14	PIK3CG	MAP2K2	MAP2K2	MAP2K2
15	PIK3R1	PIK3R2	PIK3R2	PIK3R2
16	PIK3R2	PIK3CG	PIK3CG	PIK3CG
17	PRKCA	PLCB1	PLCB1	PLCB1
18	GSK3A	PLCG1	PLCG1	PLCG1
19	GSK3B	CYP2D6|LOC101929829	CYP2D6|LOC101929829	CYP2D6|LOC101929829
20	MTOR	MAP2K1	MAP2K1	
21	MZF1	TNF	TNF	
22	MYB	RPS6KA3		RPS6KA3
23	CDKN1A		SOD2	SOD2
24	NFKB1			HFE
25	CDKN2B			MAP2K1|SNAPC5
26	TNF			CDKN2B-AS1|CDKN2B
27	SOD2			PLCG1|MIR6871
28	HFE			PLCG1|MIR6871
29	SLC29A2			
30	RPS6KA3			
31	CYP2D6			

Red genes are not mutated at baseline nor at the 4th cycle (T4) or the 8th cycle (T8) of Lenalidomide and Azacitidine therapy
